# Altered PI3-Kinase/Akt Signalling in Skeletal Muscle of Young Men with Low Birth Weight

**DOI:** 10.1371/journal.pone.0003738

**Published:** 2008-11-17

**Authors:** Christine B. Jensen, Malgorzata S. Martin-Gronert, Heidi Storgaard, Sten Madsbad, Allan Vaag, Susan E. Ozanne

**Affiliations:** 1 Steno Diabetes Center, Gentofte, Denmark; 2 University of Cambridge Metabolic Research Laboratories, Institute of Metabolic Science, Cambridge, United Kingdom; 3 Department of Endocrinology, Hvidovre University Hospital, Copenhagen, Denmark; Ordway Research Institute, United States of America

## Abstract

**Background:**

Low birth weight (LBW) is associated with increased future risk of insulin resistance and type 2 diabetes mellitus. The underlying molecular mechanisms remain poorly understood. We have previously shown that young LBW men have reduced skeletal muscle expression of PI3K p85α regulatory subunit and p110β catalytic subunit, PKCζ and GLUT4 in the fasting state. The aim of this study was to determine whether insulin activation of the PI3K/Akt and MAPK signalling pathways is altered in skeletal muscle of young adult men with LBW.

**Methods:**

Vastus lateralis muscle biopsies were obtained from 20 healthy 19-yr old men with BW</ = 10^th^ percentile for gestational age (LBW) and 20 normal birth weight controls (NBW), matched for physical fitness and whole-body glucose disposal, prior to (fasting state) and following a 4-hr hyperinsulinemic euglycemic clamp (insulin stimulated state). Expression and phosphorylation of selected proteins was determined by Western blotting.

**Principal Findings:**

Insulin stimulated expression of aPKCζ (p<0.001) and Akt1 (p<0.001) was decreased in muscle of LBW men when compared to insulin stimulated controls. LBW was associated with increased insulin stimulated levels of IRS1 (p<0.05), PI3K p85α (p<0.001) and p110β (p<0.05) subunits, while there was no significant change in these proteins in insulin stimulated control muscle. In addition LBW had reduced insulin stimulated phospho-Akt (Ser 473) (p<0.01), indicative of reduced Akt signalling. Insulin stimulated expression/phosphorylation of all the MAPK proteins studied [p38 MAPK, phospho-p38 MAPK (Thr180/Tyr182), phospho-ERK (Thr 202/Tyr204), JNK1, JNK2 and phospho-JNK (Thr 183/Tyr185)] was not different between groups.

**Conclusions:**

We conclude that altered insulin activation of the PI3K/Akt but not the MAPK pathway precedes and may contribute to development of whole-body insulin resistance and type 2 diabetes in men with LBW.

## Introduction

Developmental programming is the concept whereby nutritional or environmental stimuli, acting during critical windows in development, may have a lasting impact on cellular structure and function, and consequently, on patterns of disease [1]. Low birth weight (LBW), a surrogate marker of an adverse fetal environment, is associated with development of insulin resistance and type 2 diabetes [2]. However, the underlying molecular mechanisms remain poorly understood.

Skeletal muscle accounts for the majority of insulin-stimulated glucose disposal [3], and defects in muscle insulin action represent an early marker for diabetes risk [4]. Healthy individuals with LBW have reduced muscle mass [5] and have recently been demonstrated to have altered fibre composition in vastus lateralis muscle [6], implying a role for skeletal muscle in the pathogenesis of insulin resistance in LBW. Moreover, young LBW men with normal glucose tolerance and normal whole-body glucose disposal have reduced forearm (muscle) glucose uptake following acute local insulin infusion [7] and decreased fasting expression of key insulin signalling proteins in vastus lateralis muscle [8], further supporting this concept.

Insulin signalling is mediated by a highly complex network controlling a variety of different processes. Briefly, in the presence of insulin, the insulin receptor phosphorylates insulin receptor substrate (IRS) proteins, which are linked to the activation of two main signalling pathways: the metabolic phosphatidylinositol 3 kinase (PI3K)/ protein kinase B (Akt, also known as PKB) pathway, responsible for most of insulin's metabolic actions, and the mitogenic Ras-mitogen-activated protein kinase (MAPK) pathway, which is involved in mediating cell growth, survival and differentiation [9]. We have previously shown that young LBW men have reduced skeletal muscle expression of PI3K p85α regulatory subunit and p110β catalytic subunit, atypical protein kinase C ζ (aPKCζ) and glucose transporter (GLUT4) in the fasting state [8]. These changes precede the development of whole body insulin resistance and glucose intolerance. However, it remains to be determined whether the adverse fetal environment leads to alterations in the expression of proteins in MAPK signalling pathway. Insulin can activate three major members of the MAPK family, including p38 MAPK [10], [11], c-jun NH2-terminal kinase (JNK) [12] and extracellular signal-regulated kinase 1/2 (ERK1 and ERK2) [9]. Studies have indicated that p38 MAPK is required for full GLUT4 translocation [13] and aberrant p38 MAPK signalling in skeletal muscle has been described in type 2 diabetic patients [10]. JNK activity is increased in insulin-resistant states such as obesity and inflammation and can negatively regulate insulin signalling through serine phosphorylation of IRS-1 and impaired activation of Akt [14]. ERK1 and ERK2 may also be involved in a negative feedback loop of insulin action by phosphorylating IRS-1 on serine residues [9].

Thus, the goal of this study was to determine whether altered insulin activation of the PI3K/Akt and MAPK signalling pathways could contribute to the development of insulin resistance and type 2 diabetes in LBW humans. To that end, we recruited 20 healthy 19 year old men with LBW and 20 normal birth weight (NBW) controls. All subjects had normal glucose tolerance and whole-body insulin sensitivity and were matched for physical fitness. Muscle biopsies were obtained prior to and following a 4-hour hyperinsulinemic euglycemic clamp and expression, and phosphorylation of key insulin signaling proteins was determined by Western blotting.

## Materials and Methods

### Subjects and study protocol

Forty singleton men born at term (39–41 weeks) in 1980 in Copenhagen County were recruited from the Danish Medical Birth Registry according to birth weight, as previously described [15]. Twenty men had birth weights </ = the 10th percentile (LBW: 2702±202 g) and 20 men had birth weights in the upper normal range (NBW: 50–75th percentile) (3801±101 g). None of the participants had a family history of diabetes (parents, grandparents), hypertension, or ischemic heart disease. All participants had normal glucose tolerance, as assessed by standard 75-gram OGTT, according to World Health Organization criteria. Subjects meeting these criteria were included consecutively and we made no specific attempts to match groups for body composition etc. Written informed consent was provided from all participants and approval was obtained from the regional ethics committee. All subjects had DEXA assessment of body composition and assessment of aerobic fitness using a submaximal VO_2_max test, as previously described [15]. On the study day, following a 24 hr standardized diet and exercise protocol, a 2-hr basal period and a standard 30-min intravenous glucose tolerance test, all subjects underwent a 4-hr two-step hyperinsulinemic euglycemic clamp (2 hrs at 10 mU/m^2^/min (low peripheral hyperinsulinemia) and 2 hrs at 40 mU/m^2^/min (prandial-like hyperinsulinemia) in combination with tritiated glucose tracer and indirect calorimetry, as previously described [15]. Muscle biopsies were excised at the end of the basal (+2 hrs after initiation of study) and insulin-stimulated steady state period (+4 hrs after initiation of insulin infusion).

### Muscle Biopsies

Percutaneous muscle biopsies were obtained from the vastus lateralis muscle during local anaesthesia using a Bergstrøm needle. Samples were blotted free of blood, connective tissue and visible fat, snap-frozen in liquid nitrogen and stored at −80°C until further analysis.

### Western blotting

Muscle biopsies were extracted in ice-cold lysis buffer [50 mmol/l HEPES (pH 8), 150 mmol/l sodium chloride, 1% Triton X100, 1 mmol/l sodium orthovanadate, 30 mmol/l sodium fluoride, 10 mmol/l sodium pyrophosphate, 10 mmol/l EDTA and a protease inhibitor cocktail]. The total protein concentration in the lysates was determined using a Sigma copper/bicinchoninic assay. Protein content of muscle tissue did not differ between any of the groups. Samples were diluted to a common concentration of 1 mg/ml in Laemmli buffer and 20 ug total protein was subjected to SDS-PAGE. The proteins were transferred to PVDF Immobilon-P (Millipore) membrane, blocked for 1 hr (5% nonfat dehydrated milk, 1×TBS, 0.1% Tween 20), followed by overnight incubation with antibody against IRS-1 and PI3K p85α (Upstate Biotechnology, Lake Placid, USA); Akt1, Akt2, phospho-Akt (Ser473) (all Cell Signalling Technology, Beverly, USA); PKCζ, PI3K p110β (Santa Cruz Biotechnology, Santa Cruz, USA), phospho-ERK1/2 (Thr202/Tyr204), p38 MAP-kinase, phospho-p38 MAP-kinase (Thr180/Tyr182), JNK1, JNK2 (all Cell Signalling Technology, Beverly, USA); phospho-JNK (Thr183/Tyr185) and GLUT4 (both Abcam, Cambridge, UK) diluted in TBS–0.1% Teen 20 containing 5% dried milk or 5% BSA. Protein expression was quantified densitometrically using AlphaEase software (AlphaInnotech, San Leandro, USA). 20 µg and 10 µg of one sample was loaded onto each gel in order to ensure the linearity of the signal and to act as an inter-gel control.

Due to the limited size of the biopsy two strategies were used to analyse protein expression. The first strategy was used for the analysis of proteins that have not been previously analysed under basal conditions: IRS-1, Akt2, phospho-Akt, p38 MAPK, phospho-p38 MAPK, JNK1, JNK2, phospho-JNK, phospho-ERK1. Three gels were used for the analysis of each protein, each gel containing six samples randomly chosen from the four study groups. For the analysis of PI3K p85α, PI3K p110β, PKCζ, Akt1 and GLUT4, which have been analysed under basal conditions previously, insulin-stimulated NBW and LBW muscle samples were run on two gels alongside 6 basal NBW samples (mean % control +/−1 STD) chosen on the basis of the previous study (8). For each antibody, control blots were performed in which varying amounts of protein (5 µg, 10 µg and 20 µg) were loaded onto the gel to ensure that the chemiluminescent signal changed in a linear manner. Primary and secondary antibody concentrations were also optimized.

### Statistical analyses

Differences between individual baseline characteristics were analyzed by Student's t-test or non-parametrical tests (Wilcoxon, Mann-Whitney) when appropriate (Table 1). Nonparametric data were log transformed prior to testing and are shown as the % geometric mean basal control (95% confidence intervals). Parametric data are represented as % mean basal control±SEM. Differences in insulin stimulation between the two birth weight groups were analyzed using a two-way repeated measurement ANOVA with birth weight and insulin-stimulation as the independent variables, followed by Duncan's Post Hoc test when appropriate (Statistica 7.1, Statsoft, Tulsa, USA). For all data sets, a p*-*value <0.05 was considered statistically significant.

**Table 1:** Baseline Characteristics

	LBW Mean±SD	NBW Mean±SD	p value
N	20	20	
Birth weight (g)	2702±202	3801 ±101	**<0.0001**
Height (cm)	178.5±4.0	181.7±4.8	**0.03**
Weight (kg)	73.6±8.5	74.7±13.1	NS
BMI (kg/m^2^)	23.1±2.7	22.6±3.6	NS
W/H-ratio	0.82±0.04	0.81±0.06	NS
Total lean mass _DEXA_ (kg)	54.9±4.3	56.9±7.3	NS
Total fat mass _DEXA_ (kg)	15.5±6.8	15.6±6.9	NS
Abdominal fat mass _DEXA_ (kg)	33.1±18.1	30.7±21.9	NS
VO_2_max (L/mol)	3.4±0.4	3.5±0.7	NS
f-pl-glc (mmol/L)	5.6±0.4	5.4±0.4	**0.05**
f-pl-ins (pmol/L)	56.1±39.6	48.6±22.2	NS
Rd _40mU_ (mg/kg FFM/min)	11.1±3.1	11.9±3.0	NS
Rd _40mU_ (mg/kg FFM/min)	3.9±2.6	5.4±2.6	**0.04**

Differences between individual baseline characteristics were analyzed by Student's t-test, or non-parametrical tests (Wilcoxon, Mann-Whitney) when appropriate. p*-*value <0.05 was considered statistically significant.

## Results

### Clinical characteristics (Table 1)

Demographic and metabolic data [15], fibre composition [6] and expression of select proteins in vastus lateralis muscle in the fasting state [8] have been reported previously. These studies showed that 19-yr old men with LBW (<10^th^ percentile) had comparable current body weight, BMI, W/H-ratio, VO_2_max and total lean and fat mass with the NBW men, but were slightly shorter and had a tendency toward more central fat accumulation (as shown by the increased ratio of abdominal fat mass/total fat mass p<0.05). LBW was associated with a higher proportion of “glycolytic” type 2× (formerly 2b) fibres at the expense of fewer but larger “oxidative” type IIa fibres. In addition, the LBW men had slightly higher fasting plasma glucose and reduced insulin-stimulated whole-body glycolytic flux but normal whole-body glucose disposal, glucose oxidation, non-oxidative glucose metabolism and energy expenditure in the fasting and insulin-stimulated state.

### Protein expression-basal (fasting) state (Fig. 1 and Table 2)

**Figure 1:**
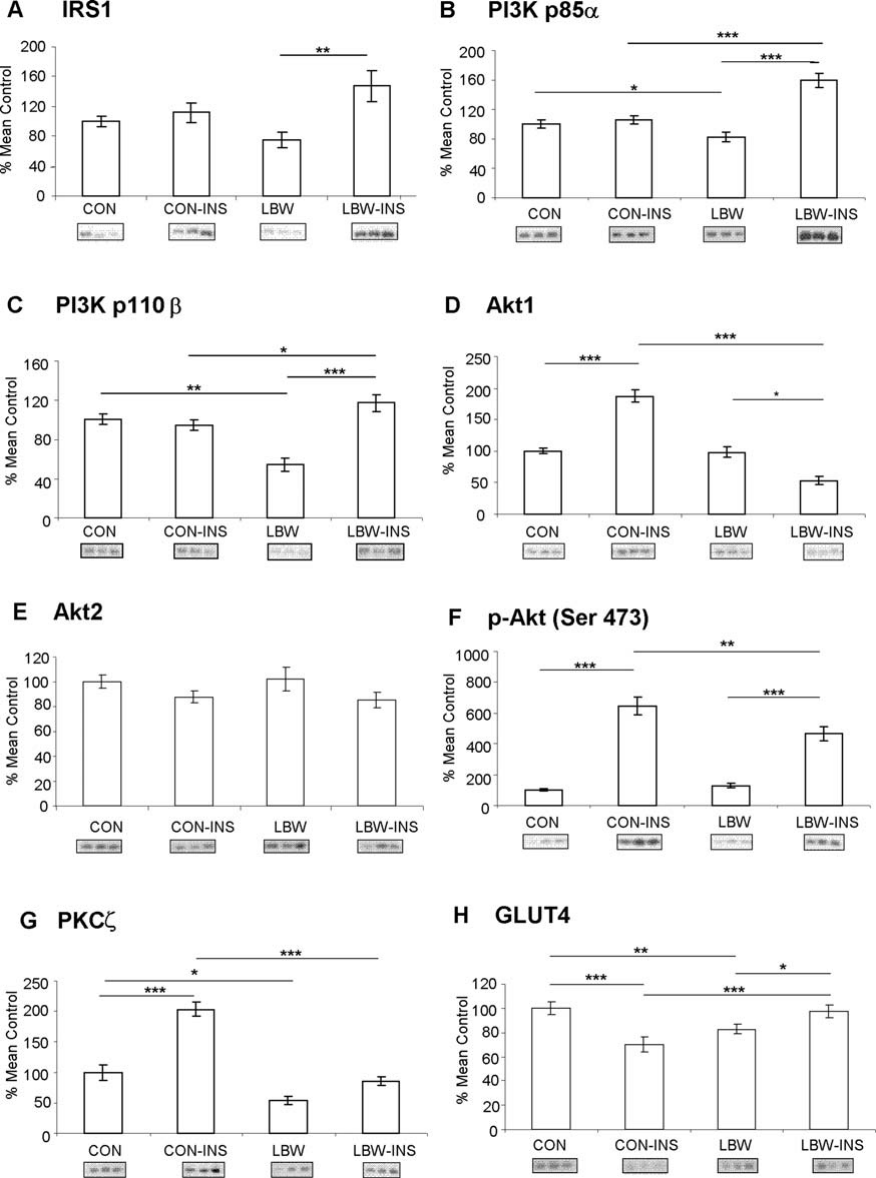
Expression and phosphorylation of key proteins involved in PI3K signalling. Bars represent mean basal and insulin-stimulated (INS) expression values±SEM for low birth weight (LBW) and control (CON) individuals, expressed as % of mean control. Representative blots of the respective proteins are located below the corresponding bar graphs. *p<0.05, **p<0.01, ***p<0.001.

Fasting expression of the PI3K p85α and p110β subunits, PKCζ and GLUT4 was reduced in LBW subjects when compared to controls, as previously reported (8) (Figure 1). Expression/phosphorylation of other proteins studied involved in metabolic pathway [(IRS1, Akt1, Akt2 and phospho-Akt (Ser 473)] or mitogenic pathway [p38 MAPK, phospho-p38 MAPK (Thr180/Tyr182), JNK1, JNK2, phospho-JNK (Thr 183/Tyr185) and phospho-ERK (Thr202/Tyr204)] was unchanged in LBW subjects in fasting state in comparison to the controls [Figure 1 and Table 2].

**Table 2:** Expression and phosphorylation of key proteins involved in MAPK-signalling.

Protein expression/phosphorylation	Control	Control Insulin stimulated	LBW	LBW Insulin stimulated
p-ERK (Thr202/Tyr204)	100 [15–228]	117 [26–303]	129 [35–349]	119 [49–407]
p-p38 (Thr180/Tyr182)	100 [11–220]	61 [0–262]	71 [5–182]	46 [32–247]
p38	100±8	102±8	110±17	107±14
p-JNK (Thr183/Tyr185)	100±9	87±10	100±11	109±9
JNK1	100±7	135±14	117±14	119±7
JNK2	100±7	104±8	104±9	95±6

Values are presented as the geometric mean basal control [95% confidence limits] for p-ERK (Thr202/Tyr204) and p-p38 (Thr180/Tyr182) and as the % mean basal control±SEM for p38, p-JNK (Thr183/Tyr185), JNK1 and JNK2. Statistical differences were analysed using a repeated measures ANOVA. Data for p-ERK (Thr202/Tyr204) and p-p38 (Thr180/Tyr182) were log transformed prior to testing.

### Protein expression–insulin-stimulated state (Fig. 1)

#### (1) IRS1 and PI3K

Insulin infusion (4 hrs) *per se* did not lead to changes in expression of IRS1 in muscle of control subjects however LBW demonstrated enhanced expression for IRS-1 under insulin stimulated conditions (p<0.01) (interaction between birth weight and presence of insulin p<0.01) (Fig. 1A). Similarly, expression of PI3K p85α (Fig. 1B) and p110β (Fig. 1C) did not change upon insulin infusion in controls but despite lower basal (fasting) expression LBW men had significantly increased p85α (p<0.001) and p110β (p<0.001) after insulin infusion (interaction between birth weight and insulin infusion p<0.001 for both PI3K subunits). In addition the expression of both PI3K subunits was significantly increased in insulin stimulated LBW subjects in comparison to the insulin stimulated controls (for p85α p<0.001 and for p110β p<0.05).

#### (2) Akt

There was an interaction between birth weight and insulin infusion on Akt1 expression (p<0.001). Insulin infusion significantly increased expression of Akt1 (p<0.001) in control subjects but it decreased expression of Akt1 in LBW men (p<0.05) (Fig. 1D). No differences were observed between groups in Akt2 expression (Fig. 1E). There was an interaction between birth weight and insulin infusion on phosphorylation of Akt at Ser 473 (p<0.05) (Fig. 1F). Insulin infusion significantly increased phosphorylation of Akt (Ser 473) in both control (*p*<0.001) and LBW groups (*p*<0.001), however the effect was greater in controls when compared to the LBW men (p<0.01).

#### (3) PKCζ and GLUT4

There was an interaction between birth weight and insulin infusion on PKCζ expression (p<0.001). Insulin infusion significantly increased expression of PKCζ in control subjects (p<0.001), however no significant difference was observed in LBW subjects (Fig. 1G). There was significant reduction in PKCζ expression in LBW men upon insulin stimulation when compared to insulin stimulated control subjects (p<0.001). Interestingly, insulin infusion had very different effect on GLUT4 expression. Insulin infusion lead to reduction in expression of GLUT4 (p<0.001) (Fig 1H) in controls, while significant increase occurred in LBW men (p<0.05). Despite lower basal (fasting) expression (p<0.01), insulin-stimulated expression of GLUT4 was increased in LBW men in comparison to controls (p<0.001). Interaction between birth weight and insulin stimulation on GLUT4 expression was significant (p<0.001).

#### (4) Mitogenic proteins

There were no differences in the expression/phosphorylation of p38 MAPK, phospho-p38 MAPK (Thr180/Tyr182), JNK1, JNK2, phospho-JNK (Thr 183/Tyr185) and phospho-ERK (Thr202/Tyr204) (Table 2).

## Discussion

We have previously shown that young and healthy, low birth weight men, exhibit multiple abnormalities in their skeletal muscle in the insulin signalling metabolic pathway downstream of the insulin receptor, under fasting conditions [8]. In this study we aimed to investigate whether any further alterations in either mitogenic (MAPK) or metabolic (PI3K/Akt) pathways downstream of insulin receptor can be detected in skeletal muscle in response to *in vivo* insulin stimulation that could explain why are LBW men at higher risk of developing insulin resistance and type 2 diabetes in later life. The main finding of this study is marked insulin-mediated up-regulation in LBW subjects of the PI3K p85α and p110β subunits and reduced expression/phosphorylation of its two main effectors, PKCζ and phospho-Akt at Ser 473, both of which are required for insulin-stimulated glucose uptake [9], [16].

Previously, we have reported reduced abundance of p85α, p110β, PKCζ and GLUT4 proteins in skeletal muscle of LBW men under fasting conditions [8]. Similar reductions of p85α, p110β and GLUT4 were observed in subcutaneous abdominal fat biopsies from an independent LBW cohort, together with a marked reduction of IRS-1 [17]. IRS molecules are key mediators of insulin signalling and play a central role in maintaining basic cellular functions such as growth, survival and metabolism. Using siRNA (small interfering RNA)- mediated knockdown of IRS proteins, it was found that IRS-1, rather than IRS-2 is required for insulin-stimulated Akt1 phosphorylation, GLUT4 translocation and glucose uptake [18], [19]. Here we show that skeletal muscle IRS-1 expression was similar in control and LBW men. This is consistent with previous reports of normal IRS1 expression in the skeletal muscle from diabetic subjects [20]. We also show that insulin-stimulated expression of IRS-1 was increased in LBW men but not in controls. Recent studies in L6 myotubes showed that, prior to its down-regulation by degradation, IRS-1 protein is acutely induced by insulin stimulation [21]. Thus, the inappropriately increased IRS-1 in LBW at 4 h of insulin stimulation may be a consequence of delayed/protracted stimulation and/or delayed degradation.

Despite lower expression in the basal state, insulin stimulation increased expression of PI3K subunits p110β and p85α in LBW subjects but not in controls. The mechanistic basis of this paradoxical increase following insulin infusion is unknown. Increased expression of p85α has been implicated in insulin resistance as knockout of p85α improves insulin sensitivity *in vitro* [22] and *in vivo* [23]. Conversely, over-expression of p85α is correlated with skeletal muscle insulin resistance in obesity and type 2 diabetes [24] and has been reported in insulin-resistant states induced by e.g. growth hormone excess [25] and short-term overfeeding [26]. It is thought that excess p85α may exert these effects by sequestration of IRS-1 and PI3K enzymatic activity into inert cellular foci incapable of PI-3, 4, 5-triphosphate (PIP_3_) generation [27].

PI3K is thought to mediate many of its metabolic actions of insulin through phosphorylation of Akt. Phosphorylation of Akt at Ser473 was reduced in the LBW group compared to controls following insulin infusion, suggesting impaired PI3K activity. Limited sample availability meant that actual activity could not be determined. Decreased insulin stimulated Akt kinase activity has been previously reported in type 2 diabetic subject [28], [29] and non-obese spontaneously diabetic Goto-Kakizaki (GK) rats [30]. As well as a reduction in PI3K activity, reduced Akt1 expression could also contribute to reduction in phospho-Akt detected in the insulin stimulated LBW muscle. However, Akt2 is the prominent isoform in skeletal muscle [31], [32] thus phosphorylated Akt detected primarily represents phospho-Akt2. Our findings of no difference in the basal and insulin stimulated expression of Akt2 between control and LBW confirm previous finding that insulin stimulation has no effect on total Akt expression [29]. Knockout and siRNA studies have revealed that whereas Akt2 is indispensable for glucose homeostasis, Akt1 is essential for growth and may play a role in lipid metabolism [9], [33]. Decreased Akt1 levels in LBW may reflect a general resistance to stimulation by growth factors and combined with our findings in the basal state, could contribute to the reduced muscle mass observed in LBW men.

In addition to decreased phosphorylation of phospho-Akt we have also found significant reduction in the expression of PKCζ in LBW subjects. Activation of PKC isoforms λ and ζ is required for GLUT4 translocation and glucose uptake in the insulin stimulated state [34]–[36]. This is supported by reports showing that over-expression of a dominant-negative mutant of PKCζ in muscle cells leads to abrogation of insulin-stimulated glucose transport and GLUT4 translocation [37], [38], while over-expression of PKCζ in skeletal muscle *in vivo* enhanced both basal and insulin-stimulated glucose transport [39]. The importance of PKCζ in pathogenesis of insulin resistance *in vivo* has been suggested by numerous studies showing impaired activation of PKCζ in skeletal muscle in obese subjects [40], impaired glucose tolerance [41], type 2 diabetes [40], [41] and in a rat model of intrauterine growth restriction (IUGR)[42]. Here we show that LBW men have diminished basal and insulin stimulated expression of PKCζ, which would provide less substrate for full activation.

GLUT4 levels, despite being lower under basal conditions were paradoxically increased by insulin infusion in LBW men. Thus, while insulin-stimulation reduced total GLUT4 in the control subjects, consistent with two previous reports [43], [44], no such effect was observed in LBW men. Interestingly, a similar lack of response to insulin was also observed in type 2 diabetes patients [43] and following insulin resistance induced by lipid infusion [44], indicating that this phenomenon may indeed be related to insulin resistance. Consistent with our previous observations in humans [8], it was recently shown that adult rat IUGR offspring have reduced GLUT4 abundance in muscle in the basal state [45]. Moreover, redistribution of GLUT4 from intracellular compartments to the plasma membrane in the basal state was partly responsible for a total inability of GLUT4 to respond to insulin [45]. Thus, it is possible that the higher insulin-stimulated GLUT4 protein levels observed in our LBW subjects do not translate into increased GLUT4 translocation and improved glucose disposal.

Following reports of aberrant p38 MAPK signalling in skeletal muscle of type 2 diabetic patients [10] and increased phosphorylation and activity of p38 MAPK upon hyperglycaemia in cultured L6 myotubes [46] we decided to asses whether insulin action upon mitogenic signalling pathway can be also implicated in the pathogenesis of type 2 diabetes in LBW men. Insulin stimulated expression/phosphorylation of all the MAPK proteins studied p38 MAPK, phospho-p38 MAPK (Thr180/Tyr182), phospho-ERK (Thr 202/Tyr204), JNK1, JNK2 and phospho-JNK (Thr 183/Tyr185) was not different between NBW and LBW groups studied. Therefore we can conclude that key proteins involved in MAPK signalling pathway do not contribute to alterations observed in skeletal muscle of young LBW men [6]. It is possible that the changes reported in p38 MAPK signalling in type 2 diabetic patients are a consequence of diabetes and may develop in LBW as their glucose homeostasis deteriorates.

In summary, we have demonstrated that LBW, a known risk factor for development of insulin resistance and type 2 diabetes, is associated with multiple defects in the PI3K/Akt pathway under basal conditions and following *in vivo* insulin-stimulation. Given that these were young healthy subjects, matched for total body fat, VO_2_max and whole-body insulin sensitivity, we speculate that these are primary defects, likely to contribute to the pathogenesis of skeletal muscle loss and development of muscle insulin resistance associated with LBW. Prospective studies are needed to establish the significance and/or reversibility of these findings.
